# Ecosystem Health Responses of Urban Agglomerations in Central Yunnan Based on Land Use Change

**DOI:** 10.3390/ijerph191912399

**Published:** 2022-09-29

**Authors:** Binpin Gao, Yingmei Wu, Chen Li, Kejun Zheng, Yan Wu

**Affiliations:** 1 Faculty of Geography, Yunnan Normal University, Kunming 650500, China; 2 Yunnan Academy of Social Sciences, Kunming 650000, China

**Keywords:** land use change, ecosystem health, ecosystem services, InVEST model, urban agglomerations

## Abstract

Land use change in urban agglomerations is gradually becoming a major cause and a key factor of global environmental change. As a consequence of the interaction between land use and ecological processes, the transformation in natural ecosystem structure and function with human activity disturbances demands a systematic assessment of ecosystem health. Taking the Central Yunnan urban agglomeration, undergoing transition and development, as an example, the current study reveals the typical land use change processes and then emphasizes the importance of spatial heterogeneity of ecosystem services in health assessment. The InVEST model-based ecosystem service assessment is incorporated into the ecosystem health evaluation, and hotspot analysis is performed to quantitatively measure the ecosystem health response degree to land use according to spatial latitude. The study had three major findings: First, the urban land expansion in the urban agglomeration of central Yunnan between 1990 and 2020 is the most significant. Further, the rate of the dynamic change of urban land is 16.86%, which is the highest among all land types. Second, the ecosystem health of the central Yunnan urban agglomeration is improving but with obvious spatial differences, showing a trend of increasing from urban areas to surrounding areas, with the lowest ecosystem health level and significant clustering in the areas where the towns are located. The ecosystem health level is mainly dominated by the two classes of ordinary and well grades, and the sum of the two accounts for 63.35% of the total area. Third, the process of land transfer, mutual transfer between forest and grassland, and conversion from cropland to forest land contributed the most to the improvement of ecosystem health across the study area. Furthermore, the conversion from cropland and grassland to urban land is an important cause of the sustained exacerbation of ecosystem health. Significantly, the study provides a scientific reference for maintaining ecosystem health and formulating policies for macro-control of land in the urban agglomerations of the mountain plateau.

## 1. Introduction

With the process of globalization and integration of the world economy, international competition and cooperation relations have expanded from single urban cities to urban agglomerations [1]. The spatial structure of China’s economic development has also profoundly changed, and urban agglomerations have become the main spatial form for bearing development factors and gradually become an important spatial unit for urbanization construction in China [2]. In recent years, China’s major urban agglomerations, such as the Yangtze River Delta, the Pearl River Delta, and the Beijing–Tianjin–Hebei urban agglomerations, have been developing rapidly. The 19 national urban agglomerations that have been formed carry more than 75% of the urban population and contribute more than 80% of the country’s GDP, but they have also been sensitive areas where ecological and environmental problems are highly concentrated and exacerbated. Urban agglomerations in China face serious problems caused by the imbalance between urbanization and ecosystem interaction processes [3,4], which is directly manifested by the dramatic changes in land use patterns, resulting in the degradation of ecosystems and their service functions, significantly altering the health and integrity of natural ecosystems. In the face of the current situation, countries around the world are paying more and more attention to the far-reaching effects of land use changes caused by rapid urbanization on global ecosystems [5,6], it is important to assess the ecological effects of land use changes from the perspective of ecosystem health [7,8,9] to coordinate the relationship between economic development and ecological conservation in urban agglomerations areas.

Ecosystem health refers to maintaining the integrity, stability, and sustainability of ecosystem structure and function with disturbances due to human activity and is considered the ultimate goal of environmental management [10,11,12]. A healthy ecosystem should be active, maintainable in its organizational structure, and able to recover itself under stress, and it is the core guarantee of sustainable human development [13,14]. Most existing studies assessing ecosystem health used either the indicator system approach [15,16,17], the pressure-state-response (PSR) framework [18,19,20], or the vigor-organization-resilience (VOR) framework [21,22]. However, to evaluate ecosystem health from the landscape viewpoint, exploring the interaction between ecosystem service change and different land use types is important, besides considering the changes in landscape structure due to human activities [23]. Based on this, Xiao et al., Ge et al., Xu et al. and Wang et al. [24,25,26,27] all explored the effects of land use change from an ecosystem health perspective and explained the ecological effects of regional land use change from different aspects.

Unlike economically developed urban agglomerations in China, the Central Yunnan urban agglomerations, as one of the important urban agglomerations in Southwest China, is at the stage of transition from early development to maturity. Although the urban agglomerations in central Yunnan have rich biological resources and innate advantages for development, their ecosystem is more sensitive. In recent decades, with constant frictions between natural ecosystems and urban construction, makes it an ideal research area for studying land use change and ecosystem health responses. In addition to incorporating the InVEST (Integrated Valuation of Ecosystem Services and Tradeoffs) model-based assessment of ecosystem service into the ecosystem health framework, this study further explores spatially the response of two trends of ecosystem health-deterioration and improvement of the study area to land use change. The key objectives of the study include: (1) To study the features of land use change of the Central Yunnan Urban Agglomeration between 1990 and 2020. (2) To assess the ecosystem health in the central Yunnan urban agglomerations and analyze its spatial and temporal evolutionary characteristics. (3) To quantitatively measure land use change responses in the deteriorating and improving ecosystem health areas. The study significantly contributes to government policies and decision-making in formulating land management plans, balanced development, and meeting conservation needs. Meanwhile, it also provides a reference for the enrichment of the ecosystem health evaluation system for mountainous urban agglomerations.

## 2. Materials and Methods

### 2.1. Study Area

**Figure 1:**
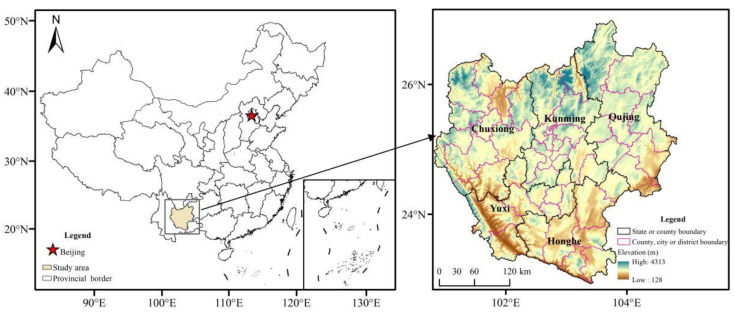
Location of central Yunnan urban agglomeration in China.

The central Yunnan urban agglomeration is situated in the central Yunnan Province in southwest China (24°58′ N–25°09′ N, 100°43′ E–104°49′ E) (Figure 1). It is the most economically developed region in Yunnan Province, including 49 counties, cities, and districts, with a land area of 111,356.04 km^2^, accounting for 28.26% of the province’s land area, of which only 11.84% is plains. The study area belongs to the lake basin landscape of the Zhongshan plateau, with karst landforms developed in the east. The overall mountain and inter-mountain basin topography are dominant, with a large vertical height difference (between 116~4282 m above sea level), which is a typical plateau mountainous urban agglomeration. Due to the complex topographic fragmentation and fragile ecological environment in the region, it is among the urban agglomerations of western China that are more seriously constrained by topographic structures, resources, and environment. The main climate type is low latitude plateau mountain monsoon climate with a small annual temperature difference but significant vertical differences in climate, rainfall increases with altitude, good lighting conditions, and rich biological resources.

As a major growth pole of China’s Yangtze River Economic Belt, the central Yunnan urban agglomeration is in a transitional stage from the nurturing phase of development to the mature phase. With a population of over 21 million, accounting for 46.5% of the province’s population, and a total GDP exceeding 1500 billion yuan, accounting for 61.47% of the province’s GDP. The dense transportation facilities, rapid population, and economic and social development of the cluster area have caused the large-scale transformation of ecological land into construction land. Additionally, due to the high-intensity economic development, the ecological and environmental problems brought about by the continuous expansion of the urban scale have become more and more prominent.

### 2.2. Data Sources

The research adopts 30 m resolution land use data of 1990, 2000, 2010, and 2020 as the basis for the study. The dataset comes from the Resource and Environmental Science Database of the Chinese Academy of Sciences (http://www.resdc.cn) (accessed on 6 October 2021), with Landsat5/7/8, GF2 and other satellite remote sensing images as the main information sources. And according to the research objectives and the actual situation of the surface, the land use types are divided into seven categories, which included cultivated land, forest land, grassland, water area, urban land, rural land, and unutilized land. Other physical, geographic, and economic data include elevation, precipitation, soil depth, potential evapotranspiration, normalized vegetation index, net primary productivity, grain yield, and sown area. Table 1 shows the specific sources for each of these data types used in the research.

### 2.3. Methodological Steps

**Figure 2:**
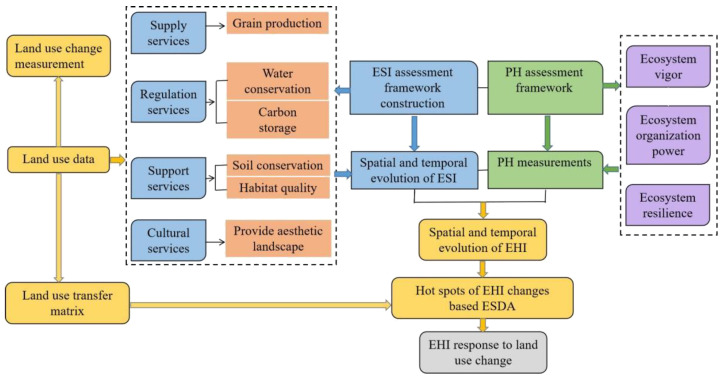
Flowchart of methodological steps. ESI (ecosystem service index), PH (physical health index), EHI (ecosystem health index).

Firstly, the spatial and temporal characteristics of land use types in the central Yunnan urban agglomeration area from 1900 to 2100 were analyzed by land use change measurement. Second, the InVEST model was used to assess changes in grain production, water conservation, carbon storage, soil conservation, habitat quality, and provide aesthetic landscape. This was applied to the physical health level assessment of ecosystems quantified by the vigor–organization–resilience (VOR) model, which together form a framework for ecosystem health assessment. Finally, the exploratory spatial data analysis (ESDA) method was used to explore the “cold spots” and “hot spots” of ecosystem health changes in the whole central Yunnan urban agglomeration. The response of ecosystem health to land use change was further revealed. The specific flowchart is as follows (Figure 2):

#### 2.3.1. Land Use Change Measurement

(1) The land use dynamics degree. It can quantitatively describe the quantity change in a certain land use type at a certain time. The land use change process across the study area can be observed by calculating the dynamic degree of every land use type, using the following calculation formula:
  $$D_{i}=\left(\frac{S_{i}^{t2}-S_{i}^{t1}}{S_{i}^{t1}}\right)\times \frac{1}{\Delta t}\times 100\%\;$$
  where *D* represents the dynamic attitude in land type *i*th, $S_{i}^{t2}$ suggests the area in land type *i* at the latter moment, $S_{i}^{t1}$ means the area in land type *i* at the initial moment, Δt refers to the time interval, and if the time interval is measured in years then *D* denotes the average annual change rate in land type *i*.
(2) Land use type transfer. The land use data across the study area was calculated by two-by-two superposition through the raster calculation in ArcGIS 10.8 to conclude the land use type transfer and spatial distribution across the study area in two phases with the following formula:
  $$C_{i^{*}j}=A_{i^{*}j}\times 10+B_{i^{*}j\;\;\;}\;(\mathrm{Applicable}\;\mathrm{when}\;\mathrm{land}\;\mathrm{use}\;\mathrm{type}\;<\;10)$$
  where $C_{i^{*}j}$ refers to the land use change from period *A* to period *B*, and $A_{i^{*}j}$ and $B_{i^{*}j}$ are the land use types of any two periods.

#### 2.3.2. Ecosystem Health Assessment Framework

Costanza proposed that understanding ecosystem health requires the recognition of humans as an important component of ecosystems [11]. Traditional ecosystem health assessments explore the sustainability of spatial unit patterns and ecological processes only in terms of the ecosystem itself, ignoring the benefits that humans derive from a properly functioning ecosystem [29], whereas ecosystem services can precisely link ecosystem processes, functions, and human well-being.

Therefore, this study follows the ecosystem health assessment framework proposed by Costanza and draws from the “ecosystem vigor-ecosystem organization-ecosystem resilience-ecosystem services” integrated ecosystem health assessment framework constructed by Peng et al., Pan et al. and Chen et al. [30,31,32]. To further emphasize the importance of spatial heterogeneity of ecosystem services in health assessment, we incorporated ecosystem service indicators quantified using the InVEST model into the assessment framework in an attempt to enhance the richness of ecosystem health assessment indicators and further improve the scope and precision of the assessment perspective [14]. The evaluation of the ecosystem health in the central Yunnan urban agglomeration comprises two components: the physical health level in the ecosystem and the integrated ecosystem service index. The specific formula is as follows:

$$EHI=\sqrt[{}]{PH\times ESI}\;$$

where *EHI* means the ecosystem health in the assessed area; *PH* denotes the physical health index in the assessed area; *ESI* is the integrated ecosystem service index of the assessed area.

#### 2.3.3. Selection and Assessment of Integrated Ecosystem Service Indicators

The UN Millennium Ecosystem Assessment categorized the ecosystem service function classification system into four major functions: product supply service, regulating service, supporting service, and cultural service. Among them, product supply service function is the function of the ecosystem to produce or provide products; regulating service function is the function of the ecosystem to regulate human ecological environment including water production capacity and carbon storage capacity; supporting service function is the basic function necessary to ensure the provision of all other ecosystem service functions, including soil conservation, habitat quality, and cultural service function is the aesthetic landscape experience, non-material benefits from the ecosystem [33].

For assessment, six typical ecosystem service functions, namely, habitat quality, carbon storage, soil conservation, water conservation, grain production, and provide aesthetic landscape, were selected from the four major services, including ecosystem supply services, regulation services, support services, and cultural services. These ecosystem service functions are consistent with the typical features of the central Yunnan urban agglomeration and reflect the integrated water, food, soil, atmospheric, and overall ecological information of the habitat. The details of the specific process are shown in Table 2.

To comprehensively measure the ecosystem services in the central Yunnan urban agglomeration, the evaluation results for the above six ecosystem service functions were normalized in ArcGIS. After eliminating the effects by different magnitudes, they were superimposed and calculated according to the mean weights. The integrated Ecosystem Service Index (ESI) in the central Yunnan urban agglomeration in 1990, 2000, 2010, and 2020 were each calculated as follows.

$$ESI=\sum_{i=1}^{n}\;W\times ES^{\prime }\;$$

where *ESI* is the integrated ecosystem service index for the study area; *W* means the weight coefficient for various ecosystem service types; *ES’* is the standardized function of different types of ecosystems, and *n* is the type of ecosystem services in this study (*n* = 6).

#### 2.3.4. Ecosystem Physical Health Indicator Selection and Assessment

The physical health of the ecosystem can be assessed on the basis of three criteria: the activity of the ecosystem to provide energy, the structure of the ecosystem to maintain health under stress, and the ability of the ecosystem to self-regulate and recover. This study is based on the results of Das, Manob et al. [38] and Pan et al. [32] to characterize the ecosystem’s physical health with three indicators of health level: vigor (V), organization (O), and recovery (R).

(1) Ecosystem vigor (V): The NPP bands of 2000, 2010, and 2020 were cut and spliced by obtaining MODIS data products to calculate the annual true values of NPP for the four periods of the central Yunnan urban agglomeration. As the NPP reflects more vegetation vigor in terrestrial ecosystems, and the highland lakes in the study area play a key role in ecosystem vigor, the watershed was set to 1 in the normalization process according to the actual situation in the study area [39,40].

(2) Ecosystem organization power (O): The Weighted Mean Fractional Dimension (AWMPFD), Shannon Diversity Index (SDI), and Simpson Diversity Index (MSDI) were selected to measure the landscape heterogeneity (LH). Sprawl (CONTAG), separation (SPLIT), and connectivity (CONNECT) were then used to characterize landscape connectivity. In addition, woodlands and watersheds bear vital ecological functions of the central Yunnan urban agglomeration and should be protected as priority landscape types. Thus, the separation (SPLIT) and connectivity (CONNECT) of important patches were included as important landscape connectivity (ILC) separately in the calculation of ecosystem organization power. For the weight setting, landscape heterogeneity, and important landscape connectivity, weights were determined with reference to the previous studies. After each index was obtained through Fragstates software, the ecosystem organization power was calculated and normalized [23].

$$EO=0.35\times LC+0.35\times LH+0.3\times ILC\;\;=0.1\times AWMPED+0.15\times SDI+0.1\times MSDI+0.1\times CONTAG+0.25\times SPLIT\;+0.1\times \left(SPLIT_{1}+SPLIT_{2}\right)+0.05\times \left(CONNECT_{1}+CONNECT_{2}\right)$$

where *EO* means the ecosystem organization force coefficient, *LH* represents landscape heterogeneity, *LC* denotes landscape connectivity, *ILC* is important to landscape connectivity, *SPLIT_1_* and *SPLIT_2_* are the separation degree of watershed and woodland, respectively, and *CONNECT_1_* and *CONNECT_2_* are the connectivity degree of watershed and woodland, respectively.

Ecosystem resilience (R): Referring to the study by Liu et al. and Peng et al. [7,15], the resilience and resistance in various land use types were assigned. The resilience coefficients in various types of sites were corrected by combining NDVI data of the study area (Formula (1)). In the correction process, the study considered that water bodies do not have obvious vegetation reflection characteristics; therefore, water bodies were not included in the correction process to guarantee the accuracy of the results. The central Yunnan urban agglomeration is an area of intensive human activities, and the high-intensity human activities and rapid economic development have caused damage to the ecosystem caused by disturbances from outside, which have exceeded the ecosystem’s regulation capacity. Therefore, the resilience weighting should be higher than the resistance, which is set at 0.6 and 0.4, respectively (Formula (2)). The regional ecosystem resilience was calculated as follows.

(1) $$RC_{i}=\frac{NDVI_{i}}{NDVI_{-}{\mathrm{mean}}_{j}}\times RC_{j\;}\;$$

where *RC_i_* denotes the resilience coefficient of raster *i*, *NDVI_i_* denotes the *NDVI* value of raster *i*, *NDVI_mean_j_* denotes the average *NDVI* value of class *j* at raster *i*, and *RC_j_* means the resilience assignment in land use class *j* (Table 3).

(2) $$ER=0.6\times RC_{i}+0.4\times RT_{j}\;$$

where *ER* is the ecosystem resilience index for the central Yunnan urban agglomeration, *RC_i_* represents the resilience coefficient for raster *i*, and *RT_j_* is the resistance assignment of raster *j* of the land use type.

Assessment of the level of physical health of ecosystems: According to the definition of ecosystem health by Costanza, the level of physical health was calculated as follows [22]:

$$PH=\sqrt[{3}]{V\times O\times R}\;$$

where *PH* represents the ecosystem health index; *V*, *O*, and *R* mean ecosystem vigor, organization, and resilience, respectively.

#### 2.3.5. Hot Spots Analysis

Hot spots analysis (Getis-ord Gi*) is performed to recognize the distribution of hot spots and cold spots in the local space of the study area [41,42]. In order to analyze the ecosystem health response to land use change of the central Yunnan urban agglomeration from spatial latitude, the research explored the spatial clustering of ecosystem health changes and identified the hot spot areas and cold spot areas. With each stage of change, the hot spot areas are referred to as ecosystem health improvement areas and cold spot areas as ecosystem health deterioration areas.

#### 2.3.6. Measuring the Impact of Land Use Change on Ecosystem Health Hot Spots Analysis

The study overlays land use change mapping and ecosystem health change hotspot analysis mapping to analyze land use change of cold hotspot areas for ecosystem health change from 1990 to 2020. The specific method is as follows: the improvement and degradation areas for each stage of ecosystem health change are calculated and determined separately by ArcGIS 10.8. The resulting area was used as the extent of land use data extraction [43] to generate the land use transfer matrix, and the impact of each transfer type on ecosystem health is calculated. The degree of effect of land use transfer on the ecosystem was calculated using the contribution indicator [44,45] with the following equation:

$$LEI=\frac{\left(LE_{t}-LE_{0}\right)LA}{TA}\;$$

where *LEI* is the contribution of ecosystem health caused by a specific type of land use shift in the study area; *LE_0_* and *LE_t_* represent the ecosystem health indices for land use type at the beginning and the end of the change, respectively; *LA* means the total area of the land use type; *TA* denotes the total area of all land types in the study area.

## 3. Results

### 3.1. Characteristics of Land Use Change in the Central Yunnan Urban Agglomeration

Forest land was the predominant land use type during the study period, occupying over 49% of the total study area (Table 4). This is followed by grassland, with over 26% of the total area, and cultivated land, with over 20% of the total area. Finally, urban land makes up the least land use type, with less than 2% of the total area. During our course of study, we observed some general features of land use change across the area. These include, firstly, a continuous increase in the urban land and water area, secondly, a continuous decrease in the cultivated land area, and lastly, first an increase and then a decrease in the forest land and rural land area, while a decrease followed by an increase in the grassland area, whereas the area of unutilized land remained basically the same for a long time.

The urban land area shows a strikingly significant increase from 284.97 km^2^ in 1990 to 1726.02 km^2^ in 2020, with the proportion increasing from 0.26% to 1.55%. According to the dynamic attitude analysis, urban land in the central Yunnan urban agglomeration has the maximum dynamic rate of change in comparison to other land types. The rate of change was 4.45%, 7.96%, and 13.34% during 1990–2000, 2000–2010, and 2010–2020, respectively, and the highest dynamic rate of change was 16.86% during 1990–2020. Although, the cultivated land and grassland area changed with higher intensity, the dynamic rate of change was significantly lower than that of urban land.

**Figure 3:**
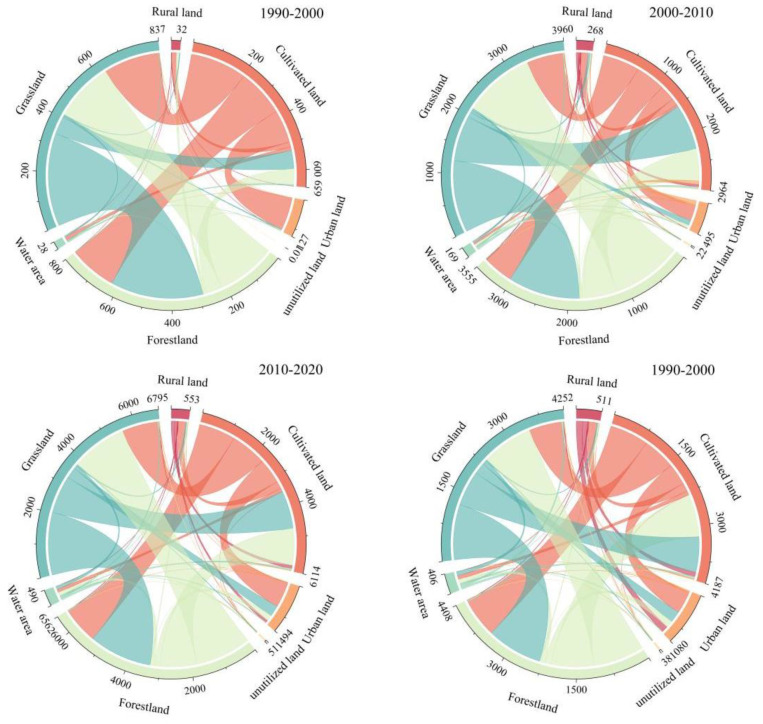
Land use change chord map of central Yunnan urban agglomeration, 1990–2020.

According to the land use transfer matrix (Figure 3), the largest area converted to urban land among all land types during 1990–2020 is cultivated land and grassland, with 818.20 km2 and 357.49 km2, respectively, accounting for 23.28% and 9.50% of the total converted area. As shown by the urban land sources at different stages, a large part of cultivated land converted, with 119.98 km^2^, 258.71 km^2^, and 476.41 km^2^ of cultivated land occupied by urban land in the three stages of 1990–2000, 2000–2010, and 2010–2020, respectively.

### 3.2. Spatial and Temporal Evolutionary Features of Ecosystem Service Functions of the Central Yunnan Urban Agglomeration

**Figure 4:**
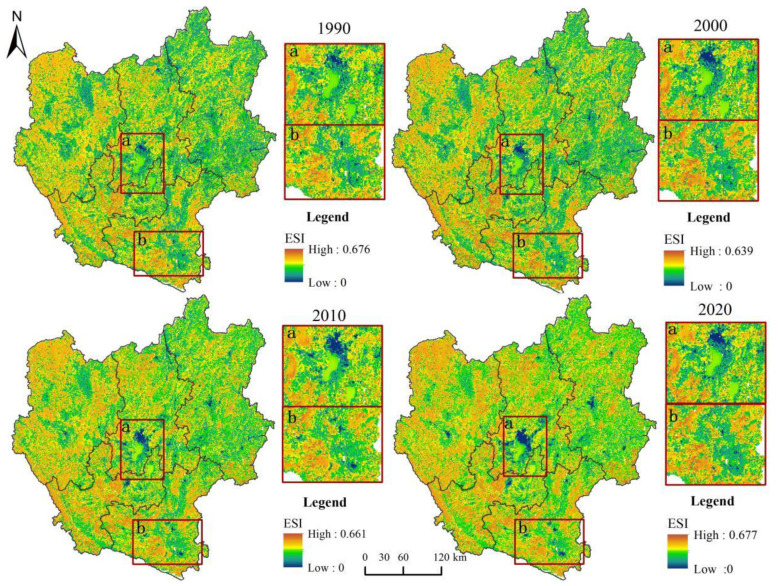
Spatial distribution pattern of ESI of central Yunnan urban agglomeration, 1990–2020.

For our research, we observed six ecosystem service functions: grain production, water content, carbon storage, soil conservation, habitat quality, and aesthetic landscape of the central Yunnan urban agglomeration. These functions were overlaid to determine the spatial distribution of the ecosystem service index (ESI) of the central Yunnan urban agglomeration in 1990, 2000, 2010, and 2020 (Figure 4). It can be seen from the figure that the ecosystem service functions of the central Yunnan urban agglomeration in 1990–2020 first decreased, then increased, and finally decreased. In 1990, the ESI values of the central Yunnan urban agglomeration were 0–0.711, with a mean value of 0.327 and an extensive distribution of low and medium values. This indicates that the ecosystem service function of the central Yunnan urban agglomeration should be promoted. The areas with high values of ESI are small and scattered, mostly in Honghe Prefecture, Yuxi and Chuxiong. For example, Jianshui County (0.362), Eshan County (0.378), and Dayao County (0.379) in these regions. The range of ESI values for the central Yunnan urban agglomeration in 2020 was 0~0.682, with a mean value of 0.3253. Although the range of high-value areas was higher compared to 1990, the frequent transformation of land use structures affected the stability of the overall ecosystem function and showed the spatial characteristics of the interactive distribution of high-value areas and low-value areas. Further, the low-value areas are primarily distributed in central Qujing City, central and southern Kunming City, central and eastern Honghe Prefecture and eastern Yuxi City. For example, Qilin District (0.269), Chenggong District (0.198), Lusi County (0.290) and Jiangchuan District (0.287) in these regions.

### 3.3. Spatial and Temporal Evolutionary Characteristics of Ecosystem Health in the Central Yunnan Urban Agglomeration

**Figure 5:**
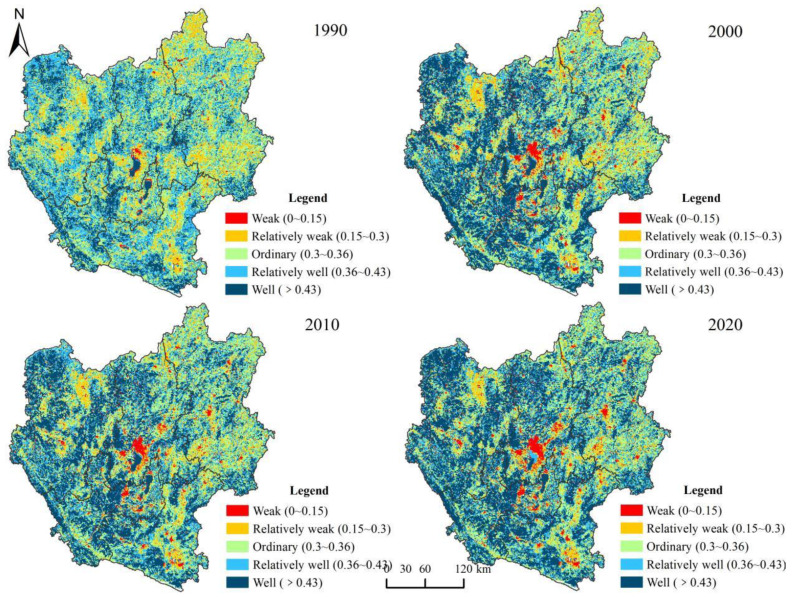
Spatial distribution of EHI by class in central Yunnan urban agglomeration, 1990–2020.

To effectively characterize the spatial differentiation of the ecosystem health index (EHI) of the study area, the natural breakpoint method was applied. Within this, the EHI of the central Yunnan urban agglomeration was divided into five classes from low to high, and the spatial distribution maps of EHI classes were obtained (Figure 5). Evidently, in the central study area, there has been a low EHI value area for a long time, i.e., the central urban area of Kunming city. Among the five levels of ecosystem health, the relatively weak and ordinary levels are mostly located in areas covered by arable land and grassland. Due to the constraints of the mountainous terrain of the study area, the arable land and grassland patches are fragmented. The land use is relatively homogeneous in these areas, making the ecosystem health of the region relatively low. The areas with high ecosystem health levels are mainly located in areas with high cover of forestland with an intensive natural ecological background.

In terms of the area ratio of ecosystem health at different levels (Table 5), in 1990, the ordinary health level accounted for 29.7% of the total area of the study area, while the relatively well health level accounted for 33.65%. The areas with well ecosystem health started to expand in 2000, wherein they accounted for 38.15% in 2020, which is an increase of 19.39% compared to 1990. Simultaneously, the areas with weak ecosystem health also expanded by 3.91%. In the future, attention needs to be paid to the possible impact on ecosystem health of a certain increase in the area of low health level.

### 3.4. Effects of Land Use Change on the Ecosystem Health of the Central Yunnan Urban Agglomeration

We observed two trends of ecosystem health changes in the central Yunnan urban agglomeration: improvement (hot spot clustering) and deterioration (cold spot clustering). After overlaying the land use change mapping with the EHI change hot spot map, the land use shifts in the colder hot spot areas in the study site between 1990 and 2020 were studied. The types of land use changes with the most significant impact on ecosystem health were obtained using the contribution ratio.

**Figure 6:**
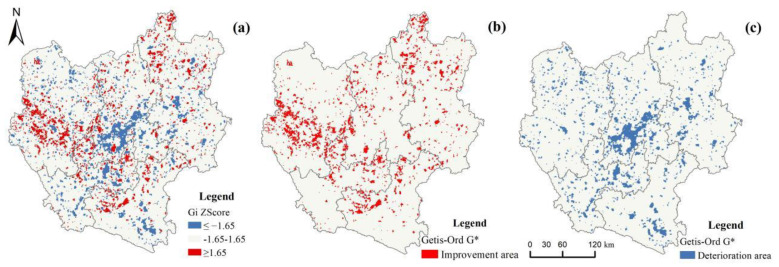
(**a**) Hot and cold spots areas of change in ecosystem health levels from 1990−2020. (**b**) Ecosystem health improvement areas. (**c**) Ecosystem health deterioration areas.

The spatial agglomeration state of EHI changes during 1990–2020 was identified by Getis-Ord G* statistics. Red areas (Gi z-score ≥ 1.65) are hot spots (ecosystem health improvement agglomerations); blue areas (Gi z-score ≤ −1.65) are cold spots (ecosystem health deterioration agglomerations); gray areas (1.65 > Gi z-score > −1.65) are non-significant zones of change (Figure 6).

**Figure 7:**
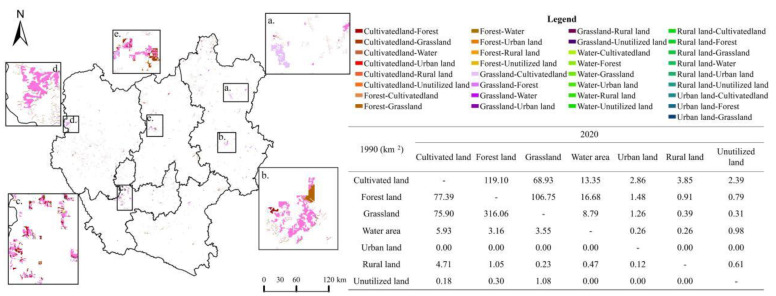
Ecosystem health improvement areas and their land use transfer matrix for 1990–2020.

**Figure 8:**
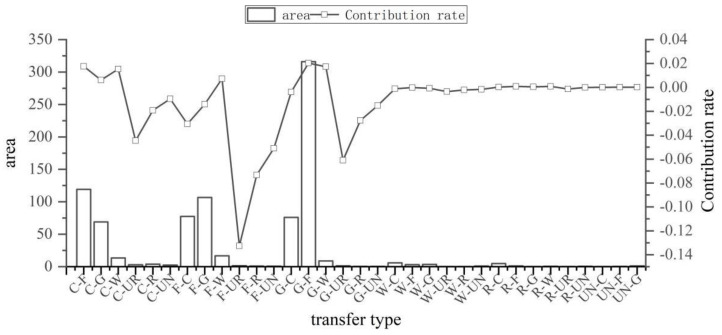
Contribution of various types of land use changes in ecosystem health improvement areas. C (cultivated land), F (forest land), G (grassland), W (water area), UR (urban land), R (rural land), UN (unutilized land).

As seen in Figure 7 and Figure 8, the types of land use changes that caused the improvement of ecosystem health from 1990 to 2020 were smaller in scale and relatively scattered. Additionally, the exchanges between forest land and grassland and the conversion from cultivated land to forest land was found to be the dominant type of land conversion across this area (a–e). The improvement in ecosystem health was driven primarily by the conversion from grassland to forest land, the conversion from cultivated land to forest land, and the conversion of grassland to the water area, with contribution indices of 0.020, 0.018, and 0.017, respectively. The shift from grassland and cultivated land to forest land provides the impetus for the continued improvement of ecosystem health.

**Figure 9:**
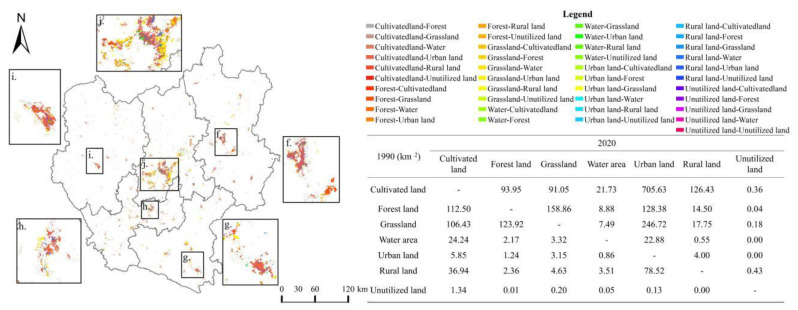
Ecosystem health deterioration areas and their land use transfer matrix for 1990−2020.

**Figure 10:**
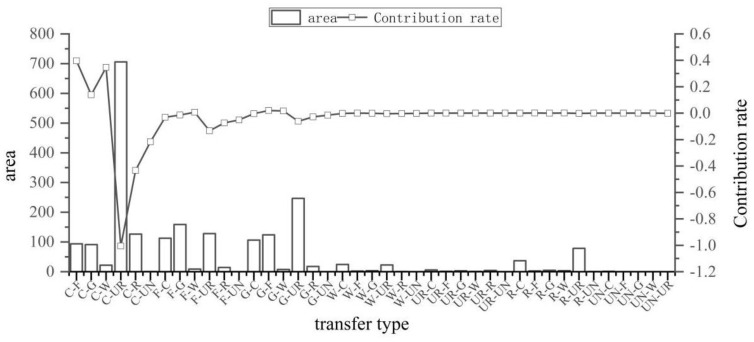
Contribution of various types of land use changes in ecosystem health deterioration areas. C (cultivated land), F (forest land), G (grassland), W (water area), UR (urban land), R (rural land), UN (unutilized land).

As seen in Figure 9 and Figure 10, the types of land use changes that caused deterioration of ecosystem health from 1990 to 2020 are relatively spatially clustered. The land use changes in the deterioration area were primarily found from the main urban area of Kunming, and the conversion from arable land and grassland to urban land was the dominant type of land use change across this area (f–j). Deterioration of ecosystem health occurred mainly due to the conversion from cultivated land to urban land, the conversion from cultivated land to rural land, and the conversion from cultivated land to unutilized land, with contribution indices of –1.04, –0.43, and –0.22, respectively. Our study determined that the deterioration of ecosystem health in the central Yunnan urban agglomeration is significantly related to land use changes due to human activities. It has caused an increasing amount of ecological land to be converted into cultivated land and urban land. It, thus, becomes a major cause of the deterioration of ecosystem health in the region.

## 4. Discussion

Our research explores the impact of land use change on ecosystem health and established that it is a key factor affecting the spatial variability of ecosystem health. We showed through our analysis that combining land use change mapping with ecosystem health hotspot analysis can effectively reveal the spatial variability of ecosystem health responses while revealing the transition patterns among various types of land uses.

### 4.1. Interpretation of Land Use Changes in Central Yunnan Urban Agglomeration

Land resources form the basic conditions and key components in the sustainable development of urban agglomerations [46]. Our study site, the urban land in the central Yunnan urban agglomeration, has seen rapid expansion since 1990. The rates of change in urban land reached 16.86% in the last 30 years, which became the most important factor perturbing the overall land structure. The central Yunnan urban agglomeration developed rapidly between 2010–2020. Further, because of the accelerated economic globalization and regional integration, four cities of Central Yunnan (Kunming, Qujing, Yuxi, and Chuxiong) signed a cooperation framework for integrated development. It resulted in the rapid economic growth, enhanced functions of the central cities, and rapid population concentration. The growth in the urbanization level, i.e., from 32.05% in 2010 to 58.94% in 2019. The conversion of forest land, grassland, and rural land into urban land has increased significantly. All four predominant cities of the central Yunnan urban agglomeration are located in the mountainous basin, and urban development is mostly concentrated in the dam area. This limits the land resources accessible to the central urban area.

Predictably, the contradictions between high concentration of population, industry, and limited land resources of the central Yunnan urban agglomeration will become the weakest link in future development. For future work on land development and management of the urban agglomeration, we should focus on characteristics like the unique natural conditions and landscape patterns of the mountainous areas, strengthening the macro control of land, improving land conservation and intensive use, and promoting land use efficiency. With guided demands and regulated supply, it will be possible to reasonably determine the new urban land scale, control the disorderly expansion of urban industrial and mining land, and guide the adjustment of the internal structure of urban land. Simultaneously, this will lead to strict control over all kinds of non-agricultural construction land occupying cultivated land, the protection of the functioning in production, ecological, and landscape isolation zones of cultivated land of the study area.

### 4.2. Ecosystem Health Level Analysis

Our study shows an improvement in the ecosystem health in the central Yunnan urban agglomeration, but the ecological health level is spatially unevenly distributed. The central and eastern regions show poorer characteristics of ecosystem health while the western region is relatively better. Among them, the areas with high values of ecosystem vigor are distributed in the areas where the lakes and waters are located, as well as the forest areas within Chuxiong, Yuxi, and Honghe (Figure S7). Driven by rapid urbanization, the spatial pattern of ecosystem health shows an increasing trend from the urban areas to the surrounding areas. This characteristic is also reflected in the sub-indices, where ecosystem organizational power shows a decreasing distribution from the central high-value area to the peripheral areas, reaching the lowest value at the edge of the study area (Figure S8). Spatial variation in ecosystem resilience coefficients is closely related to anthropogenic interventions. The lowest level of ecosystem resilience is found in areas with urban development, and the state of low-value clustering is apparent (Figure S9). This indicates that the environmental problems and landscape fragmentation, brought about by the concentration of urban population and high-intensity development, are key factors influencing ecosystem health [47].

Although the ecosystem health of the Central Yunnan urban agglomeration is relatively well, the degree of aggregation becomes more pronounced in areas of weak health. The major factors causing this include the integrated development within urban agglomerations, the amplified gravitational and spatial radiation capacity between cities, the deepening degree of interaction, and the frequent transitions between land use intensity and land use types [48]. Therefore, in the process of ecosystem health maintenance, attention should be paid to monitoring the ecological health of low-value agglomeration areas, reducing the interference of large-scale human activities on the ecosystem, overcoming the negative effects caused by the agglomeration effect, and enhancing the self-regulatory ability of the area.

### 4.3. Relationship between Land Use Change and Ecosystem Health

The ecosystem health improvements and deteriorations are majorly driven by inter-conversions among land use types of the central Yunnan urban agglomeration. Among them, the land change types with the most significant impact on ecosystem health improvement were the conversions from grassland to forest land, the conversions from cultivated land to forest land, and the conversions from grassland to water areas. Thus, the most effective means to maintain the ecological health and stability of the central Yunnan urban agglomeration would include accelerating the protection and restoration of the ecological backgrounds of the improvement area, enhancing vegetation cover and biodiversity in the area, and maintaining the ecosystem vigor of forest land and water areas. On the contrary, the types of land changes that had the most significant impact on the deterioration of ecosystem health were the conversions from cultivated land to urban land, with a contribution index of −1.04, and the area of occupied cultivated land is 818.20 km^2^. However, food security is the basis of national security, and in the face of the large-scale occupation of arable land by urban land, efforts must be made to restrict land development intensity. In the future, government decisions should encourage the development of a gradient development model for the gradient development mode of mountain towns, make rational use of land resources, strengthen intensive land use, determine the optimal land use structure, and guarantee the development of urban construction while achieving effective protection of the ecological function of arable land.

It has also been found in previous studies that urban sprawl can, directly and indirectly, lead to the degradation of ecological services and contributes to the decline in the value of global ecosystem services [49,50,51]. Rural–urban migration, economic growth, all intensified urban expansion and land depletion. During this period, the degraded areas of urban ecosystem service function in central Yunnan were also mainly concentrated in the urban expansion areas (Figures S1–S6). Therefore, in order to promote ecosystem health, policy makers should pay more attention to the contradiction between the scarcity of urban land in the study area and ecological conservation in mountainous areas. On the one hand, it implements the construction of major ecological projects such as “natural forest protection”, “return of cultivated land to forest and grass”, soil and water conservation, and rock desertification control [42], which actively carries out the restoration of degraded terrestrial ecosystems. In addition, according to the “Yunnan Province Ecological Function Zoning Plan” [52], the focus is on strengthening the ecological function protection and restoration of the middle reaches of the Jinsha River Basin Soil and Water Conservation Zone, the Red River Basin Soil and Water Conservation Zone, and the Pearl River Headwaters Water Conservation Zone. For areas with weaker ecosystem health, local governments must increase investment in environmental protection to improve water connotation and soil conservation capacity. This will improve the purification and self-regulation capacity of the ecosystem and reduce the vulnerability of the ecological environment in mountainous areas.

### 4.4. Limitations and Future Research

In the current research, the ecosystem services assessed, based on the InVEST model, were incorporated into the ecosystem health evaluation, which better reflects the ecological processes compared to the value-accounted ecosystem services. This methodology, to some extent, compensates for the study of land use change in the ecosystem material-energy cycle. However, it is still unclear how ecological processes triggered by land use change, and their cascading effects can simultaneously contribute to changes in ecosystem health levels [53]. Moreover, the study only focused on the unidirectional processes between land use change and ecosystem health effects, while the quantitative integration between natural feedback mechanisms and the land use change model needs further attention in the future [54].

As the urban agglomeration of central Yunnan gradually moves from development to maturity, the intensity of anthropogenic disturbances further increases. Based on the limited land resources in mountainous areas, the perspective of ecosystem health research on land use changes should pay more attention to the rational allocation of land resources. In addition, we should continue to explore the factors driving and influencing mechanisms of ecological health. These would provide the scientific basis for policymakers and implementers to establish suitable conservation measures.

## 5. Conclusions

The research was conducted on mountainous urban agglomerations in the southwest plateau of China. It aims at exploring the inherent correlation of regional ecosystem health with land use patterns and incorporates the InVEST model-based ecosystem service assessment into the ecosystem health evaluation. This methodology further emphasizes the interaction process between land use and natural ecosystems. Additionally, the study explored the responses of two trends of ecosystem health deterioration and improvement of the study area to land use change by combining land use change mapping and hotspot analysis. The findings of the study are shown below:

(1) Forest land was the predominant land use type during the study period. The transformation of cultivated and grassland to urban land is the most significant in the process of land type transformation. The rapid expansion of urban land became the most important factor in disrupting the overall land use structure change in the study area.
(2) The spatial variability of ecosystem health level is significant, with the central and eastern regions being worse and the western regions being relatively good. The areas with the lowest levels of ecosystem health are urban development areas.
(3) Ecosystem health is influenced by land use shifts. The improvement of health levels is closely related to the mutual transfer between forest land and grassland, and the conversion from cultivated land to forest land. The fast expansion of urban land caused by urbanization and the conversion from cultivated land and grassland to urban land are important reasons for the deterioration of ecosystem health.

## Figures and Tables

**Table 1:** Study data sources.

Data Type	Data Sources
Land use data	Resource and Environmental Science Data Center of the Chinese Academy of Sciences (https://www.resdc.cn/) (accessed on 6 October 2021).
Elevation data	Geospatial Data Cloud Platform (http://www.gscloud.cn) (accessed on 6 October 2021).
Precipitation data	National Earth System Science Data Center (http://www.geodata.cn) (accessed on 25 January 2022).
Potential evapotranspiration	National Earth System Science Data Center (http://www.geodata.cn) (accessed on 25 January 2022).
Soil depth	World Soil Database (HWSD) China Soil Dataset (v1.1) (http://www.cryosphere.csdb.cn) (accessed on 25 January 2022).
Normalized vegetation index (NDVI)	The NDVI data in 2000, 2010 and 2020 were generated based on the MODIS vegetation index products with a spatial resolution of 250 × 250 m and a 16-day temporal resolution obtained by the NASA Earth Observation System, using the annual maximum synthesis method. The NDVI data in 1990 was obtained from the national Earth System Science Data Center.
Net Primary Productivity (NPP)	The spatial resolution of the MODIS data product is 500 m, the band is cut and stitched, and the pixel value is multiplied by a scale factor of 0.0001 to calculate. The 1990 NPP data was gathered from Chen Pengfei, “Monthly net primary productivity 1 km raster dataset of Chinese terrestrial ecosystems north of 18° N (1985–2015)” [J/DB/OL]. Electronic Journal of Global Change Data Warehousing, 2019 [28]. (http://www.geodoi.ac.cn/) (accessed on 23 December 2021).
Grain production and sown area	According to“Yunnan Provincial Statistical Yearbook” to obtain the grain production and sown area of 49 (district) counties in each year.
Grain prices	National compilation of agricultural cost-benefit information.

**Table 2:** Principles and methods for assessing the ecosystem service functions of each ecosystem in the central Yunnan urban agglomeration.

Ecosystem Services	Ecosystem Functions	Fundamentals	Measurement Formula
Supply Services	Grain Production	Based on the linear correlation between grain yield and NDVI, grain yield was assigned according to the ratio of raster NDVI values to total NDVI values of cultivated land based on land use type [34].	$G_{i}=G_{sum}\times \frac{NDVI_{i}}{NDVI_{sum}}$ where *G_i_* is the grain yield of arable raster *i*, *G_sum_* suggests the total grain yield in the study unit, *NDVI_i_* means the *NDVI* value for arable raster *i*, and *NDVI_sum_* indicates the sum of *NDVI* values in the study unit.
Regulation Services	Water Conservation	According to the water cycle principle, the water yield is obtained by calculating parameters, including precipitation, plant transpiration, surface evaporation, root depth as well as soil depth [35]. Afterward the runoff path topography index is measured using the DEM and the runoff residence time on the grate is measured with soil permeability and surface runoff flow coefficient. The water yield is corrected to obtain the water content [36].	$\mathrm{Retention}\;=\min\left(1,\frac{249}{\;\mathrm{Velocity}\;}\right)\times min\left(1,\frac{0.9\times TI}{3}\right)\times min\left(1,\frac{\;\mathrm{Ksat}\;}{300}\right)\times \;Yield\;$ (1) where *Retention* means the water content (mm); *Velocity* refers to the flow rate coefficient; *TI* denotes the topographic index measured using Equation (2); *Ksat* indicates the soil saturation hydraulic conductivity (cm/d), measured with Equation (3); *Yield* denotes the water yield, measured using Equation (4). $TI=\mathrm{lg}\left(\frac{\;\mathrm{Drainage}\_\mathrm{Area}\;}{\;\mathrm{Soil}\_\mathrm{Depth}\;\times \;\mathrm{Percent}\_\mathrm{Slope}\;}\right)$ (2) where *Drainage_Area* represents the number of grids of the catchment area (dimensionless); *Soil_Depth* suggests the soil depth (mm); *Percent_Slope* indicates the percentage slope. In(Ksat)=20.62−0.96×In( Clay )−0.66×In( Sand )−0.46×In(OC)−8.43×BD (3) where *Ksat* denotes soil saturated hydraulic conductivity (cm/d), *Clay* indicates soil clay content (%), *Sand* represents soil sand content (%), *OC* refers to soil organic carbon content (%), *BD* means soil bulk weight (g/cm^3^). $Y_{jx}=\left(1-\frac{AET_{xj}}{P_{x}}\right)\times P_{x}$ (4) where *Y_jx_* represents the annual water yield; *P_x_* means the average annual rainfall in raster cell *x*; *AET_xj_* refers to the average annual evapotranspiration in raster cell *x* in land use type *j*.
	Carbon Storage	The average carbon density for above-ground carbon pool, below-ground carbon pool, soil carbon pool and dead organic carbon pool were calculated separately for different land types and were summed by multiplying the area in every land type by the corresponding carbon density.	$\begin{array}{c} C_{i}=C_{i,\;above\;}+C_{i,\;below\;}+C_{i,\;soil\;}+C_{i,\;dead}\\ C_{\mathrm{total}}=\overset{n}{\underset{i=1}{\sum }}C_{i}\times S_{i} \end{array}$ where *C_i_* is the *i*th land use type; *C_i,above_* signifies the above-ground carbon density in land use type *i* (t/hm^2^); *C_i,below_* suggests the below-ground biological carbon density in land use type *i* (t/hm^2^); *C_i,soil_* signifies the soil carbon density in land use type *i* (t/hm^2^); *C_i,dead_* denotes the carbon density of dead organic matter in land use type *i* (t/hm^2^), *C_total_* means the total carbon stock in the ecosystem (t); *S_i_* indicates the area in land use type *i* (hm^2^); *n* denotes the number of land use types, and *n* is 7 in this paper.
Support Services	Soil Conservation	Soil retention is obtained by measuring the difference between potential erosion and real erosion and adding it to the sediment holding capacity.	$SM_{x}=RKLS_{x}-USLE_{x}+SDR_{x}$ $RKLS_{x}=R_{x}\times K_{x}\times LS_{x}$ $ULSEx=RKLS\times C_{x}\times P_{x}$ $SR_{x}=SE_{x}\overset{x-1}{\underset{y=1}{\sum }}USLE_{y}\prod_{z=y+1}^{x-1}\left(1-SE_{x}\right)$ where *SM_x_* means the soil retention of raster *x*, *SDR_x_* means the sediment retention for raster *x*, and *SE_x_* represents the sediment retention efficiency for raster *x*. *PKLS_x_* suggests the potential soil loss for raster *x*, and *USLE_x_* and *USLE_y_* stand for the real erosion of raster *x* and its upslope raster *y*, i.e., soil erosion under vegetation cover and soil and water conservation measures, respectively. *R_x_*, *K_x_*, *LS_x_*, *C_x_*, and *P_x_* denote the rainfall erosion force factor, soil erodibility factor, topography factor, vegetation cover factor, and soil and water conservation measure factor for raster *x*, respectively.
	Habitat Quality	Generate habitat quality maps by the Habitat Quality module under the InVEST model, combining information on land cover and biodiversity threat factors.	$Q_{xj}=H_{j}\left[1-\left(\frac{D_{xj}^{z}}{D_{xj}^{z}+k^{z}}\right)\right]\;$ where *Q_xj_* means the habitat quality index for raster *x* in land use type *j*; *H_j_* suggests the habitat suitability in land use type *j*, with the value set to [0, 1]; *D_xj_* indicates the degradation of habitat for raster *x* in land use type *j*; k signifies the half-saturation constant, which takes half of the maximum degradation 0.056 (system default 0.5).
Cultural Services	Provide Aesthetic Landscape	The sown area, yield, and average price for three main crops (rice, wheat, and corn) in 49 (district) counties were used as the base data to calculate the economic value of crops per unit area. Combined with the base equivalence table of ecosystem services per unit area in the research by Xie et al. [37], the ecosystem service values of aesthetic landscapes were calculated and expressed spatially based on grid division.	$E=\frac{1}{7}\overset{m}{\underset{i=\;1}{\sum }}\frac{O_{i}P_{i}Q_{i}}{M}$ where *E* is the economic value of crop production per unit area of the study area; *i* means the crop type; *O_i_*, *P_i_* and *Q_i_* represent the sown area, yield per unit area and average price of *i* crops, respectively; *M* is the total area of three crops (rice, wheat, and corn) of the study area. $ESV=\sum \left(A_{i}\cdot VC_{i}\right)$ where *ESV* refers to the ecosystem service value of aesthetic landscape; *A_i_* means the area in land type *i*th; *VC_i_* represents the *ESV* coefficient of the aesthetic landscape in land type *i*th.

**Table 3:** Principles and methods for assessing the ecosystem service functions of each ecosystem in the central Yunnan urban agglomeration.

Type of Land Use	Cultivated Land	Forest Land	Grassland	Water Area	Urban Land	Rural Land	Unutilized Land
Ecosystem resilience	0.4	0.6	0.8	0.7	0.2	0.5	1
Ecosystem resistance	0.6	1	0.7	0.5	0.3	0.4	0.2

**Table 4:** Area and proportion of land use types in central Yunnan urban agglomeration, 1990–2020.

Type of Land Use	1900	2000			2010		2020	
	Area (km^2^)	Proportion (%)	Area (km^2^)	Proportion (%)	Area (km^2^)	Proportion (%)	Area (km^2^)	Proportion (%)
cultivated land	23,442.81	21.05	23,041.63	20.69	22,963.36	20.62	22,528.40	20.23
forest land	54,721.12	49.14	54,945.99	49.34	54,857.48	49.26	54,649.97	49.08
grassland	30,625.01	27.50	30,633.62	27.51	30,331.00	27.24	29,893.95	26.85
water area	1282.37	1.15	1294.87	1.16	1326.63	1.19	1461.55	1.31
urban land	284.97	0.26	411.78	0.37	739.61	0.66	1726.02	1.55
rural land	844.17	0.76	872.45	0.78	976.49	0.88	935.60	0.84
unutilized land	155.59	0.14	155.58	0.14	161.47	0.15	160.54	0.14

**Table 5:** Percentage area and amount of change of each class of EHI in central Yunnan urban group, 1990–2020.

Ecosystem Health Rating	1990	2000	2010	2020	1990–2000	2000–2010	2010–2020	1990–2020
Weak	0.68%	4.83%	4.25%	4.59%	4.15%	−0.59%	0.34%	3.91%
Relative weak	17.01%	11.42%	11.26%	9.85%	−5.59%	−0.16%	−1.14%	−7.16%
Ordinary	29.70%	27.38%	26.31%	24.09%	−2.32%	−1.07%	−2.23%	−5.61%
Relatively well	33.65%	23.13%	24.93%	23.12%	−10.52%	1.81%	−1.81%	−10.53%
Well	18.96%	33.24%	33.25%	38.35%	14.27%	0.01%	5.11%	19.39%

## Data Availability

The data that support the findings of this study are available from the corresponding author upon reasonable request.

## Supplementary Materials

The following supporting information can be downloaded at: https://www.mdpi.com/article/10.3390/ijerph191912399/s1.
