# Supplementary material for: Ecosystem Health Responses of Urban Agglomerations in Central Yunnan Based on Land Use Change
Source: Int J Environ Res Public Health. 2022 Sep 29;19(19):12399. doi: 10.3390/ijerph191912399 (PMC9564870; doi:10.3390/ijerph191912399)
Supplement: Supplementary file 1 [file ijerph-19-12399-s001.zip › ijerph-1879285-supplementary.pdf]

Ecosystem health responses of urban agglomerations in central Yunnan based on land use change

Supplementary material

1. Integrated ecosystem services assessment indicator: Grain Production.

The overall trend of grain production capacity in the central Yunnan urban agglomeration from 1990 to 2020 shows a continuous increase. Grain production per unit area increased from 0.48t/hm^2 in 1990 to 0.63t/hm^2 in 2020, with an increase of 31.25%. In terms of spatial distribution **(Figure S1)**, the high-value areas are concentrated in dense areas of arable land with relatively flat terrain. Among them, the Jinsha River, Nanpan River, and Yuanjiang River valley areas become the main grain supply areas.

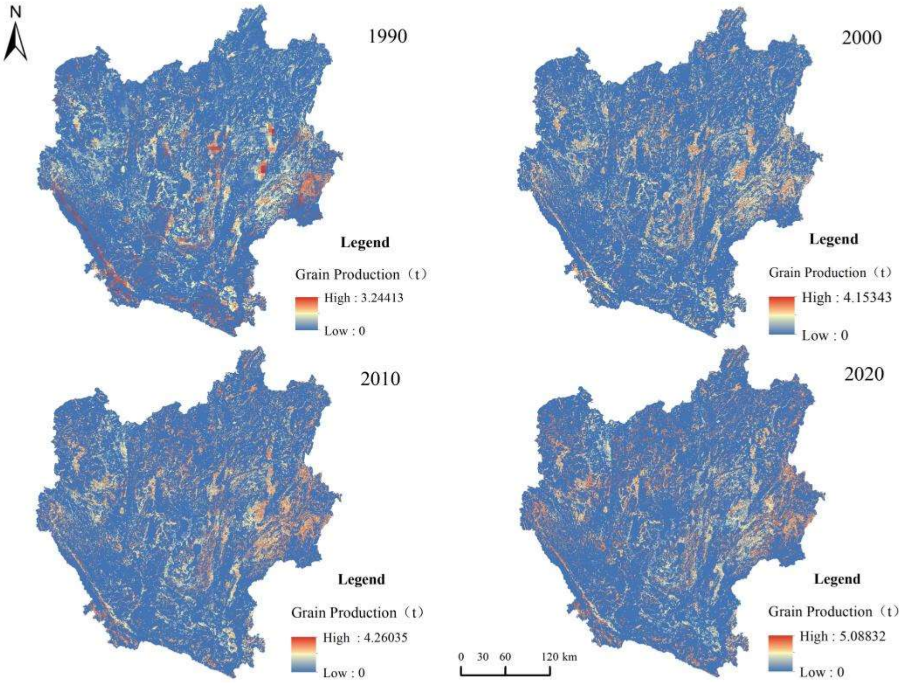

Figure S1 Spatial distribution of grain production functions in central Yunnan urban agglomeration, 1990-2020.

2. Integrated ecosystem services assessment indicator: Water Conservation.

From 1990 to 2020, the average water containment capacity and the total water containment capacity of the central Yunnan urban agglomeration showed a trend of first decreasing and then increasing. In 1990, the average water containment capacity and the total water containment capacity were 47.24mm and 52.56×10^{8}m^3 respectively; in 2000, they were 42.93mm and 47.10×10^{8}m^3 respectively; in 2010, they were 35.25mm and 39.31×10^{8}m^3 respectively; in 2020, they were 36.28mm and 40.42×10^{8}m^3 respectively **(Figure S2)**.

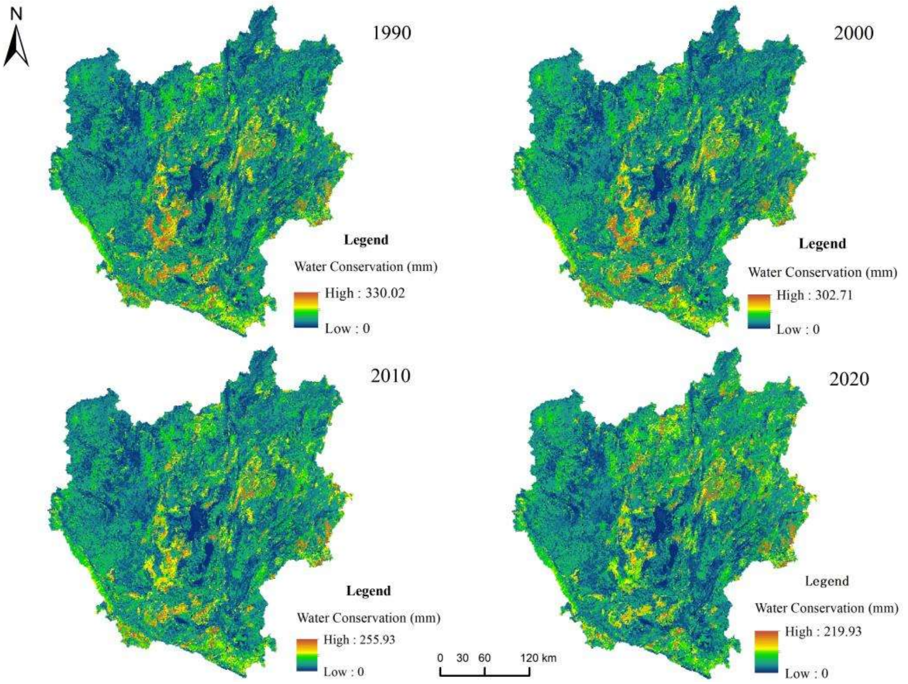

Figure S2 Spatial distribution of water conservation functions in central Yunnan urban agglomeration, 1990-2020.

3. Integrated ecosystem services assessment indicator: Carbon Storage.

In 1990, 2000, 2010, and 2020, the carbon storage capacity of Central Yunnan Urban Agglomeration is 13.761×10^8t, 13.783×10^8t, 13.754×10^8t, and 13.677×10^8t, respectively. The overall distribution of carbon storage shows that the western half of the study area is higher and the eastern half is lower, and the high-value areas of carbon storage are mainly distributed in Yongren County, Dajao County, Wuding County, Nanhua County, Lufeng County, and Shuangbai County. The land use type in these areas is mainly forested and concentrated in a continuous distribution **(Figure S3)**.

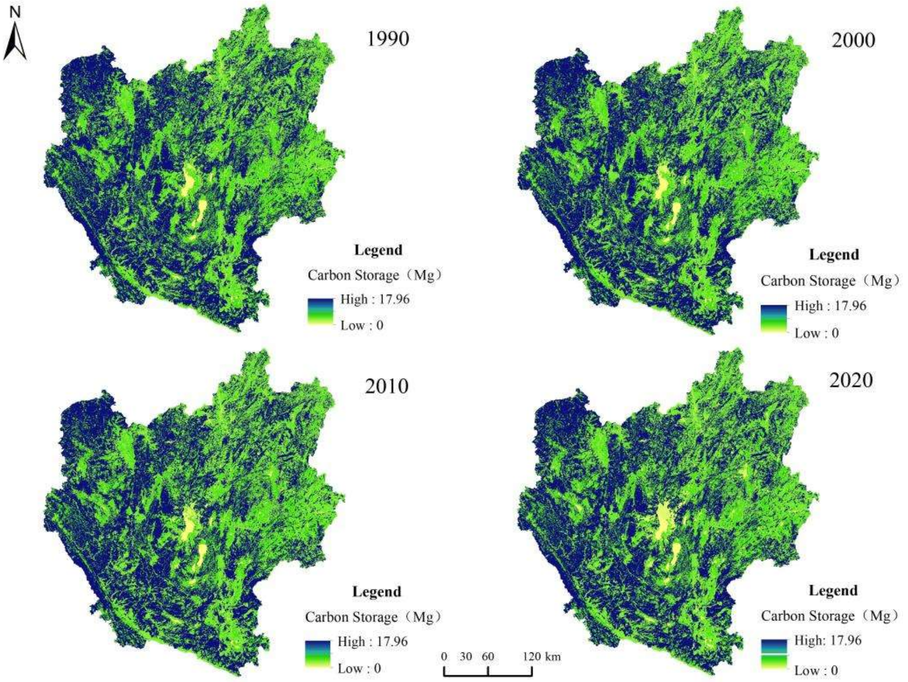

Figure S3 Spatial distribution of carbon storage functions in central Yunnan urban agglomeration, 1990-2020.

4. Integrated ecosystem services assessment indicator: Soil Conservation

From 1990 to 2020, the overall trend of soil conservation and average soil conservation intensity in the central Yunnan urban agglomeration is decreasing. in 1990, the total soil conservation in the study area was 4.89×10^9t/a and the average soil conservation intensity was 439.13t/hm^2 (soil conservation per unit area); in 2000, the total soil conservation was 4.29 $\times$ 10^9t/a, with an average soil retention intensity of 385.25t/hm^2; in 2010, the total soil retention was 3.55×109t/a, with an average soil retention intensity of 318.80t/hm^2; in 2020, the total soil retention was 3.45×10^9t/a, with an average soil retention intensity of 309.82t/hm^2. Spatially, there is a strong correlation between soil conservation capacity and topographic distribution in the central Yunnan urban agglomeration, and the areas with strong soil conservation intensity are mainly located in the western parts of Yuxi City, Chuxiong Prefecture, and Honghe Prefecture, where the topographic relief is low **(Figure S4)**.

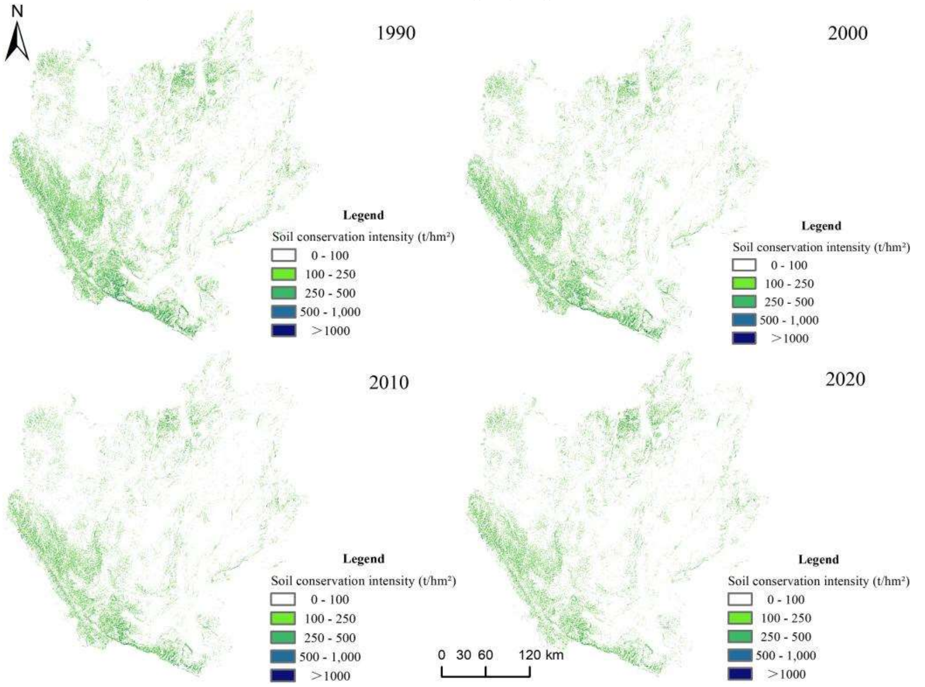

Figure S4 Spatial distribution of average soil conservation intensity in central Yunnan urban agglomeration, 1990-2020.

5. Integrated ecosystem services assessment indicator: Habitat Quality

The mean values of habitat quality in the Central Yunnan urban agglomeration in 1990, 2000, 2010, and 2020 were 0.706, 0.707, 0.715, and 0.689, respectively, showing a trend of increasing and then decreasing. In terms of spatial distribution, the habitat quality of the central Yunnan urban agglomeration shows an overall low quality of natural habitats in the urban center and surrounding areas and an increasing trend along the surrounding ring. Among them, the central cities represented by Kunming City and Lu Liang County, Qilin District, and Luxi County of Qujing City have the lowest habitat quality; the western areas in Yuxi City, Chuxiong Prefecture, and Honghe Prefecture have higher habitat quality in general **(Figure S5)**.

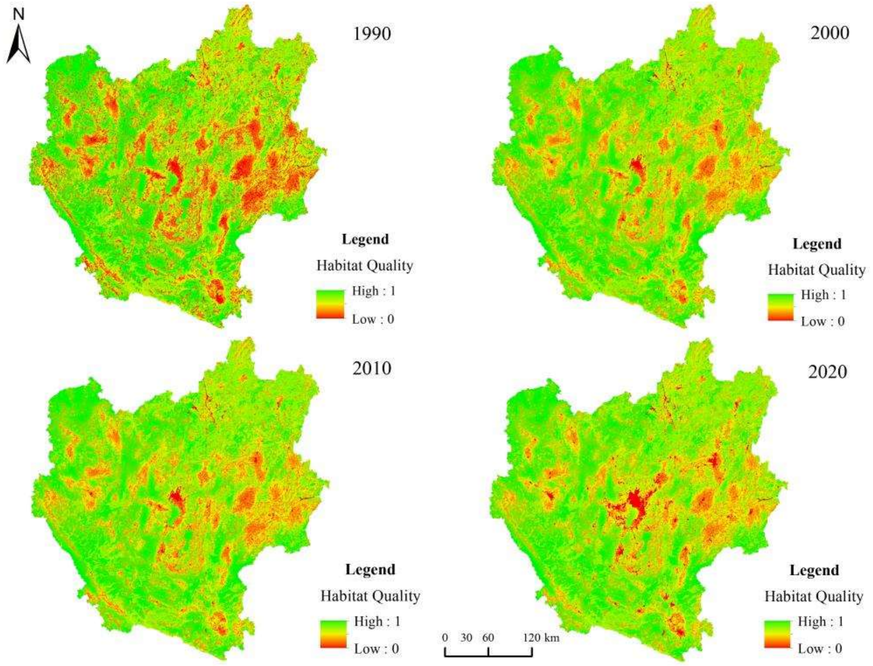

Figure S5 Spatial distribution of habitat quality in central Yunnan urban agglomeration, 1990-2020.

6. Integrated ecosystem services assessment indicator: Provide Aesthetic Landscape.

The average values of the function index of providing aesthetic landscape in central Yunnan urban agglomeration in 1990, 2000, 2010, and 2020 are 0.344, 0.345, 0343, and 0.342 respectively, showing a trend of increasing and then decreasing. In terms of each land type, areas with a large distribution of forestland provide the greatest aesthetic landscape value, followed by grassland. In contrast, cultivated land and urban land provided the lowest aesthetic value **(Figure S6)**.

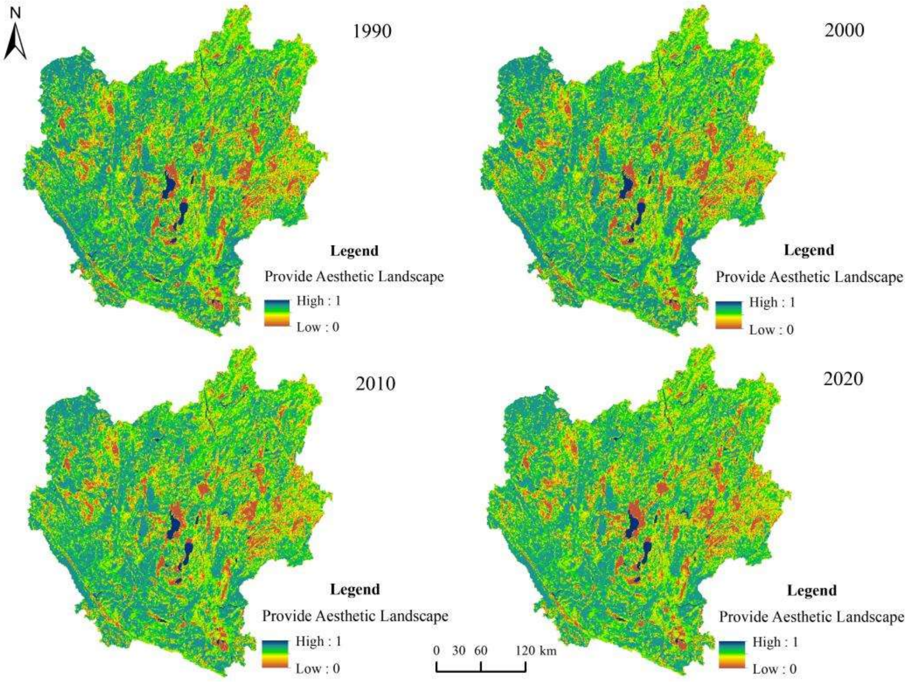

Figure S6 Spatial distribution of providing aesthetic landscape functions in central Yunnan urban agglomeration, 1990-2020.

7. Ecosystem physical health indicator: Ecosystem vigor

The mean values of ecosystem vitality of the Central Yunnan urban agglomeration in 1990, 2000, 2010, and 2020 were 0.347, 0.420, 0.414, and 0.466, respectively. In terms of spatial distribution, the range of high-value areas of ecosystem vitality in the central Yunnan urban agglomeration has been expanding since 1990, showing the characteristics of high in the southwest and low in the northeast. The high-value areas are distributed in the areas where the lakes and waters are located, as well as in the forest areas within Chuxiong, Yuxi, and Honghe. Ecosystem vitality values were lower in all areas of the urban layout **(Figure S7)**.

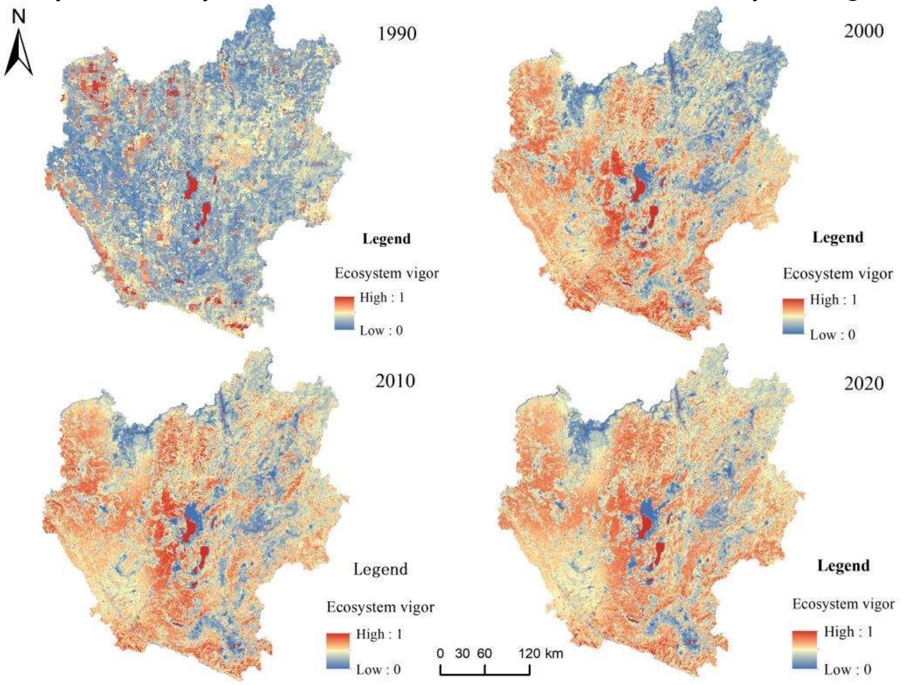

Figure S7 Spatial distribution of ecosystem vigor in central Yunnan urban agglomeration, 1990-2020.

8. Ecosystem physical health indicator: Ecosystem organization power

The mean values of ecosystem organization power in the central Yunnan urban agglomeration in 1990, 2000, 2010, and 2020 were 0.313, 0.308, 0.311, and 0.312, respectively. in terms of spatial distribution, ecosystem organization power showed a decreasing distribution characteristic from the central high-value area to the surrounding area and reached the lowest value at the edge of the study area **(Figure S8)**.

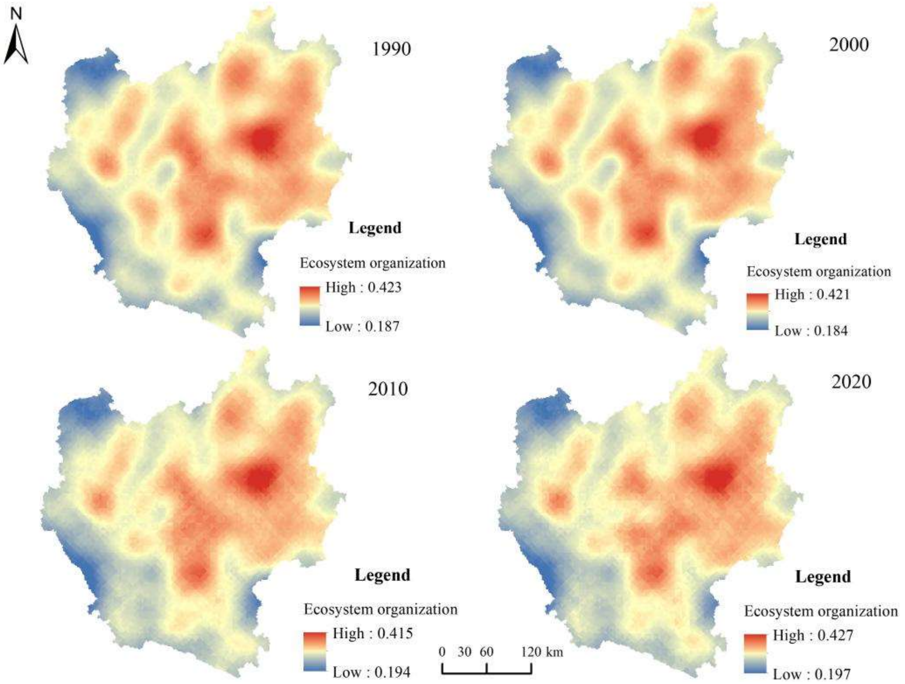

Figure S8 Spatial distribution of Ecosystem organization power in central Yunnan urban agglomeration, 1990-2020.

9. Ecosystem physical health indicator: Ecosystem resilience

The spatial variation of the ecosystem resilience coefficient in Central Yunnan urban agglomeration is closely related to the land use type. The forestland distribution areas have better vegetation conditions and a low rate of surface exposure, while the risk of intervention by human activities is low, so the ecosystem resilience coefficient is high. The low resilience coefficients were usually distributed in cultivated lands, water areas, urban and its surrounding areas **(Figure S9)**.

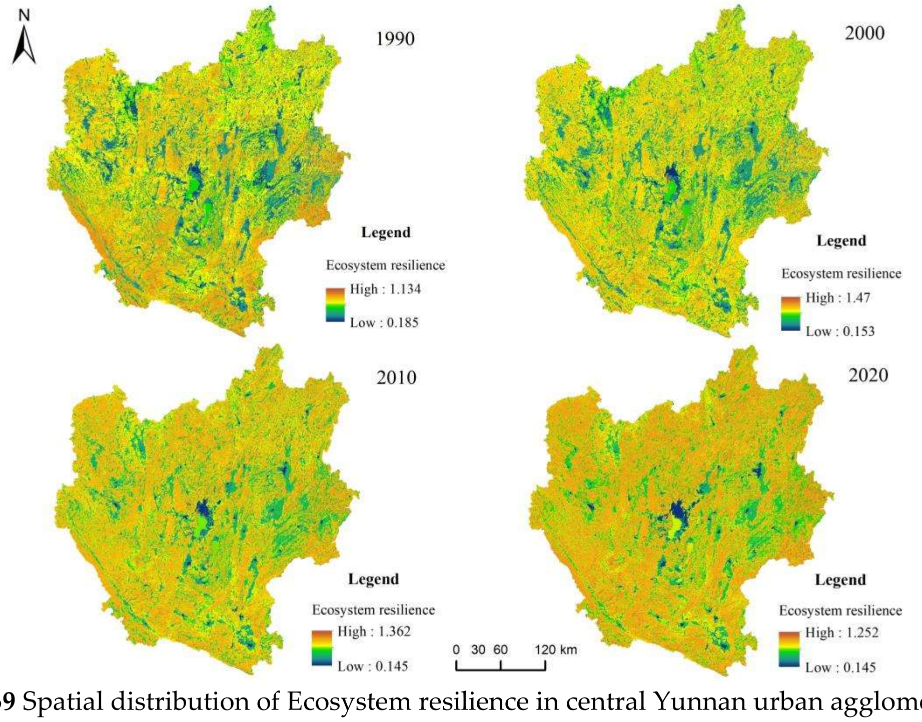

Figure S9 Spatial distribution of Ecosystem resilience in central Yunnan urban agglomeration, 1990-2020.
